# Aqueous Extract from Leaves of *Citrus unshiu* Attenuates Lipopolysaccharide-Induced Inflammatory Responses in a Mouse Model of Systemic Inflammation

**DOI:** 10.3390/plants10081708

**Published:** 2021-08-19

**Authors:** Kosuke Nishi, Takako Ito, Ayumu Kadota, Momoko Ishida, Hisashi Nishiwaki, Naohiro Fukuda, Naoaki Kanamoto, Yoko Nagata, Takuya Sugahara

**Affiliations:** 1 Department of Bioscience, Graduate School of Agriculture, Ehime University, Ehime, Matsuyama 790-8566, Japan; nishi.kosuke.mx@ehime-u.ac.jp (K.N.); ito_takako@miuraz.co.jp (T.I.); ishida.momoko.vb@ehime-u.ac.jp (M.I.); nishiwaki.hisashi.mg@ehime-u.ac.jp (H.N.); 2 Food and Health Sciences Research Center, Ehime University, Ehime, Matsuyama 790-8566, Japan; 3 Ikata Service Inc., Ikata, Ehime, Matsuyama 796-0421, Japan; a_noi@ikata-s.co.jp; 4 Ehime Institute of Industrial Technology, Matsuyama, Ehime, Matsuyama 790-1101, Japan; fukuda-naohiro@pref.ehime.lg.jp (N.F.); kanamoto-naoaki@pref.ehime.lg.jp (N.K.); nagata-youko@pref.ehime.lg.jp (Y.N.)

**Keywords:** inflammation, *Citrus unshiu*, lipopolysaccharide, RAW 264.7 cell, systemic inflammation model

## Abstract

Inflammation is related to various life-threatening diseases including cancer, neurodegenerative diseases, and metabolic syndrome. Because macrophages are prominent inflammatory cells, regulation of macrophage activation is a key issue to control the onset of inflammation-associated diseases. In this study, we aimed to evaluate the potential anti-inflammatory activity of *Citrus unshiu* leaf extract (CLE) and to elucidate the mechanism underlying its anti-inflammatory effect. We found the inhibitory activity of CLE on the secretion of proinflammatory cytokines and a chemokine from mouse macrophage-like RAW 264.7 cells and mouse peritoneal macrophages. The inhibitory activity of CLE was attributed to downregulated JNK, p38 MAPK, and NF-κB signaling pathways, leading to suppressed gene expression of inflammation-associated proteins. Oral administration of CLE significantly decreased the serum level of proinflammatory cytokines IL-6 and TNFα and increased that of anti-inflammatory cytokine IL-10 in lipopolysaccharide-induced systemic inflammation mice. In addition, oral administration of CLE decreased secretion and gene expression of several proinflammatory proteins in the liver and spleen of the model mice. Overall results revealed that *C. unshiu* leaf is effective to attenuate inflammatory responses in vitro and in vivo.

## 1. Introduction

Inflammation serves a critical role in host defense against infection by microorganisms and tissue repair [1]. Conversely, inflammation is also known to be related to various life-threatening diseases including cancer [2,3], neurodegenerative diseases [4], and metabolic syndrome [5,6]. Thus, excessive or chronic inflammatory responses should be avoided. Because macrophages are prominent inflammatory cells, regulation of macrophage activation is a key issue to control the onset of inflammation-associated diseases.

Foods with an anti-inflammatory property have recently attracted attention to prevent the incidence of inflammation-related disorders by taking them in day-to-day diet. Citrus fruits are one of the major anti-inflammatory foodstuffs [7]. Satsuma mandarin (*Citrus unshiu*), one of the citrus species, is widely distributed in Japan, Korea, China, etc. *C. unshiu* peel has been studied for its anti-inflammatory activity [8,9]; however, the potential anti-inflammatory activity of *C. unshiu* leaf has not been reported, although dried citrus leaves are used to infuse foods with citrus flavor. We thus aimed to evaluate the anti-inflammatory effect of *C. unshiu* leaf and to elucidate the mechanism underlying the anti-inflammatory effect of *C. unshiu* leaf in this study. The effects of an aqueous *C. unshiu* leaf extract on proinflammatory cytokine production and intracellular signaling in macrophages were investigated in vitro using mouse macrophage-like RAW 264.7 cells and mouse peritoneal macrophages. In addition, the in vivo effects of the extract on cytokine levels in serum and specific organs, such as the liver and spleen, were assessed using lipopolysaccharide-induced systemic inflammation mice.

## 2. Results

### 2.1. Evaluation of Cytotoxicity of CLE to RAW 264.7 Cells and Mouse Peritoneal Macrophages

**Figure 1:**
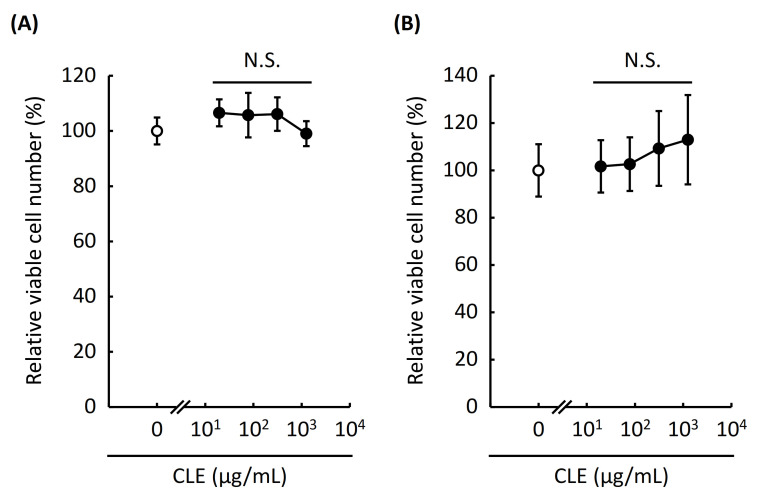
Effect of an aqueous extract from leaves of *Citrus unshiu* (CLE) on cell viability of RAW 264.7 cells and mouse peritoneal macrophages. RAW 264.7 cells and mouse peritoneal macrophages were treated with various concentrations of CLE for 24 h, followed by LPS stimulation for 6 h. Relative viable cell number was then measured using a WST-8 reagent. An open circle indicates the control cells treated with water instead of CLE. Data are represented as mean ± SEM of three independent experiments. N.S. indicates no statistical significance against control by Dunnett’s test. RAW 264.7 cells (**A**), peritoneal macrophages (**B**).

In this study, we investigated the anti-inflammatory effect of the aqueous extract from leaves of *C. unshiu* (CLE) in vitro using RAW 264.7 cells and mouse peritoneal macrophages and in vivo using a mouse model of lipopolysaccharide (LPS)-induced systemic inflammation. At first, we assessed the cytotoxicity of CLE to exclude a possibility that the anti-inflammatory effect of CLE was caused by its cytotoxicity. After treating with various concentrations of CLE for 24 h, the relative viable cell number of RAW 264.7 cells and mouse peritoneal macrophages was determined by the WST-8 assay. As shown in Figure 1A, the relative viable cell number of RAW 264.7 cells was not affected within the concentrations of CLE tested. In addition, the relative viable cell number of peritoneal macrophages was not altered by CLE either (Figure 1B). These results indicate that CLE does not affect the cell viability or proliferation of RAW 264.7 cells and mouse peritoneal macrophages. We thus proceeded to evaluating the anti-allergic effect of CLE in subsequent experiments.

### 2.2. Effect of CLE on Production of Proinflammatory Cytokines by RAW 264.7 Cells and Mouse Peritoneal Macrophages

**Figure 2:**
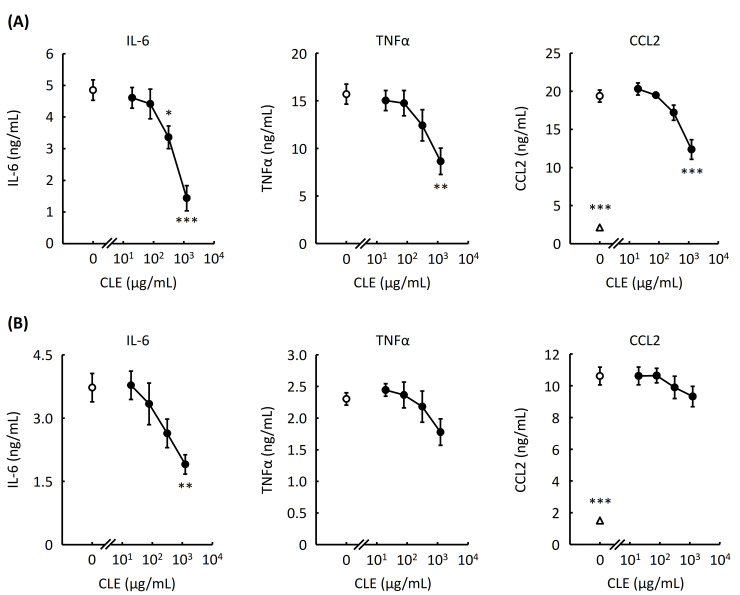
Effect of an aqueous extract from leaves of *Citrus unshiu* (CLE) on the secretion of proinflammatory proteins from RAW 264.7 cells and mouse peritoneal macrophages. RAW 264.7 cells and mouse peritoneal macrophages were treated with various concentrations of CLE for 24 h, followed by LPS stimulation for 6 h. The culture medium was subsequently used for measuring the concentrations of IL-6, TNFα, and CCL2 by ELISA kits. An open circle indicates the LPS-stimulated cells treated with water instead of CLE (control). An open triangle indicates the cells not stimulated with LPS. The concentrations of IL-6 and TNFα secreted from the cells not stimulated with LPS were below the limit of quantitation. Data are represented as mean ± SEM of three independent experiments. * *p* < 0.05, ** *p* < 0.01, or *** *p* < 0.001 against control by Dunnett’s test. RAW 264.7 cells (**A**), peritoneal macrophages (**B**).

LPS can activate RAW 264.7 cells and mouse macrophages, leading to the release of proinflammatory cytokines [10]. We thus used LPS as a macrophage activator to evaluate the anti-inflammatory effect of CLE in this study. To evaluate the effect of CLE on the release of proinflammatory cytokines, the concentrations of interleukin-6 (IL-6), tumor necrosis factor α (TNFα), and C-C motif chemokine ligand 2 (CCL2) in the culture medium of RAW 264.7 cells and mouse peritoneal macrophages were measured by enzyme-linked immunosorbent assay (ELISA) after treating with various concentrations of CLE for 24 h and subsequently stimulating with LPS. As shown in Figure 2A, CLE suppressed the secretion of IL-6, TNFα, and CCL2 from RAW 264.7 cells in a concentration-dependent manner. CLE also significantly inhibited the IL-6 production by peritoneal macrophages in a concentration-dependent manner (Figure 2B). Although statistical significance was not observed, CLE tended to suppress the secretion of TNFα and CCL2 from peritoneal macrophages.

### 2.3. Effect of CLE on Nitric Oxide Production by RAW 264.7 Cells

**Figure 3:**
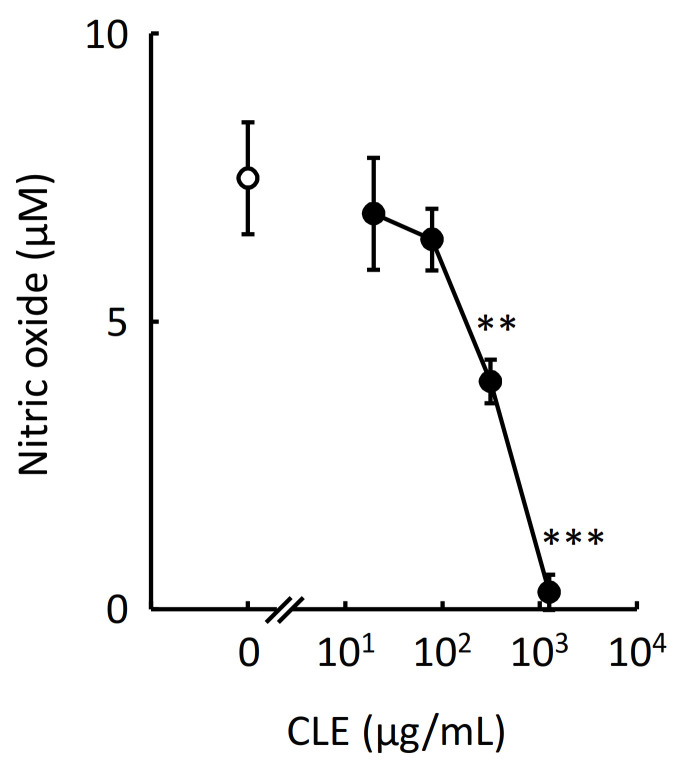
Effect of an aqueous extract from leaves of *Citrus unshiu* (CLE) on the production of nitric oxide by RAW 264.7 cells. RAW 264.7 cells were treated with various concentrations of CLE for 24 h, followed by LPS stimulation for 24 h. The culture medium was subsequently used for measuring nitric oxide concentration by Griess assay. An open circle indicates the LPS-stimulated cells treated with water instead of CLE (control). The concentration of nitric oxide produced from the cells not stimulated with LPS was below the limit of quantitation. Data are represented as mean ± SEM of three independent experiments. ** *p* < 0.01 or *** *p* < 0.001 against control by Dunnett’s test.

Activated RAW 264.7 cells produce reactive oxygen and nitrogen species, including nitric oxide, to exert the antimicrobial activity [11]; however, excess production of nitric oxide may induce a harmful response such as atherosclerosis [12]. Avoiding excessive production of nitric oxide is thus an important target in the treatment of inflammation. To evaluate the effect of CLE on nitric oxide production, we measured the nitrite concentration in this study as an indicator of nitric oxide production. After treating RAW 264.7 cells with various concentrations of CLE for 24 h and subsequently stimulating with LPS, nitrite concentration in the culture medium was measured using a Griess reagent. As shown in Figure 3, CLE significantly inhibited nitric oxide production by RAW 264.7 cells in a concentration-dependent manner.

### 2.4. Effect of CLE on the Inflammation-Related Genes Expression in RAW 264.7 Cells

**Figure 4:**
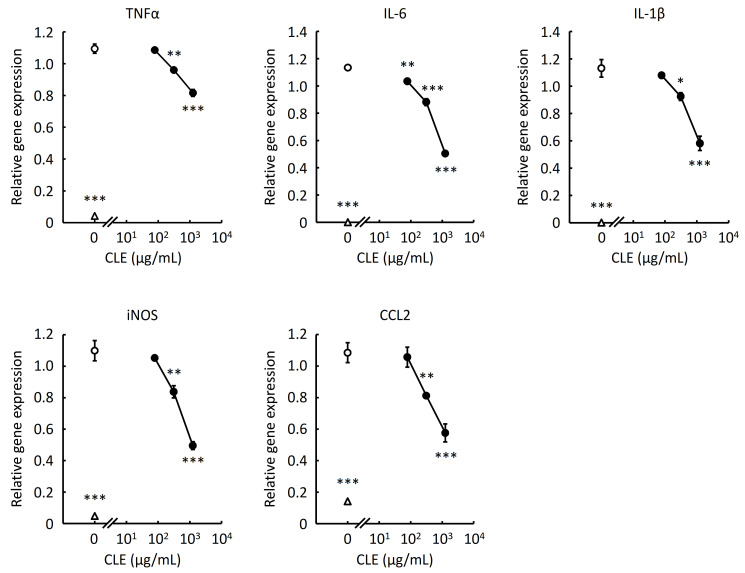
Effect of an aqueous extract from leaves of *Citrus unshiu* (CLE) on gene expression of proinflammatory proteins in RAW 264.7 cells. RAW 264.7 cells were treated with various concentrations of CLE for 24 h, followed by LPS stimulation for 24 h. Total RNA was then extracted from the cells and used for measuring relative gene expression of TNFα, IL-6, IL-1β, iNOS, and CCL2 by real-time RT-PCR. An open circle indicates the LPS-stimulated cells treated with water instead of CLE (control). An open triangle indicates the cells not stimulated with LPS. Data are represented as mean ± SEM of three independent experiments. * *p* < 0.05, ** *p* < 0.01, or *** *p* < 0.001 against control by Dunnett’s test.

Since CLE was demonstrated to inhibit production of proinflammatory cytokines and nitric oxide, we next evaluated the effect of CLE on the expression of the genes associated with inflammation in RAW 264.7 cells at the transcriptional level. As shown in Figure 4, CLE significantly inhibited the gene expression of IL-1β, IL-6, TNFα, and CCL2 in a concentration-dependent manner. The result indicates that decreased secretion of the cytokines associated with inflammation, shown in Figure 2, was attributed to downregulated transcription of the genes. CLE also significantly suppressed the gene expression of inducible nitric oxide synthase (iNOS). Because iNOS is induced by LPS to generate nitric oxide, it is highly probable that decreased production of nitric oxide, shown in Figure 3, resulted from downregulated gene expression of iNOS by CLE.

### 2.5. Effect of CLE on Phagocytotic Activity of RAW 264.7 Cells

**Figure 5:**
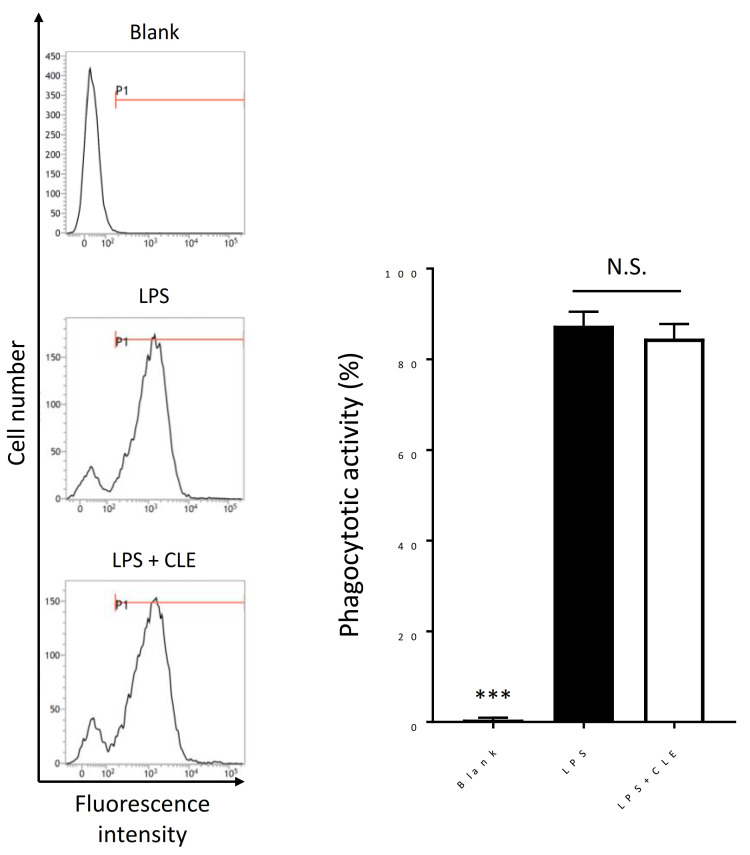
Effect of an aqueous extract from leaves of *Citrus unshiu* (CLE) on the phagocytotic activity of RAW 264.7 cells. RAW 264.7 cells were treated with 1.25 mg/mL of CLE for 24 h, followed by LPS stimulation for 1 h. The cells were next treated with Texas Red-conjugated Zymosan A for 24 h. The phagocytotic activity was then analyzed by flow cytometry. LPS indicates the LPS-stimulated cells treated with water instead of CLE, while blank indicates the cells not stimulated with LPS. Data are represented as mean ± SEM (*n* = 3). *** *p* < 0.001 against LPS by Dunnett’s test. N.S. indicates no statistical significance against LPS by Dunnett’s test.

To understand the effect of CLE on phagocytosis of macrophages, we measured the phagocytotic activity of RAW 264.7 cells using Texas Red-conjugated Zymosan A BioParticles. Phagocytosis is a critical function of macrophages for host protection and initiation of the innate and acquired immune responses. Flow cytometric analysis detected the uptake of particles following the LPS stimulation of RAW 264.7 cells (Figure 5). LPS-stimulated RAW 264.7 cells treated with CLE showed a similar phagocytotic activity to the LPS-stimulated cells without CLE treatment, indicating that CLE does not affect the phagocytotic activity of macrophages.

### 2.6. Effect of CLE on Intracellular Signaling in RAW 264.7 Cells

**Figure 6:**
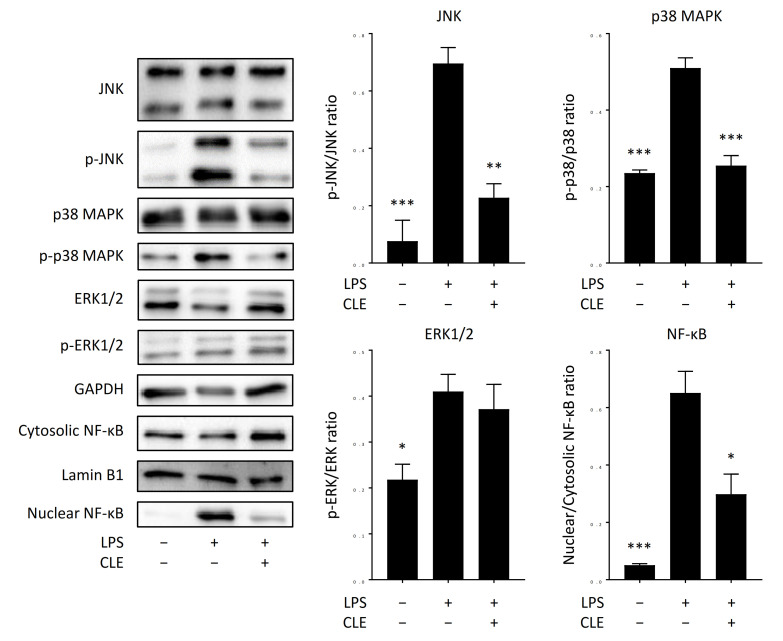
Effect of an aqueous extract from leaves of *Citrus unshiu* (CLE) on intracellular signaling pathways in RAW 264.7 cells. RAW 264.7 cells were treated with 1.25 mg/mL of CLE for 24 h, followed by LPS stimulation for 15 min. Then, the phosphorylation levels of ERK, JNK, and p38 MAPK and nuclear translocation level of NF-κB were evaluated by immunoblot analysis. A representative blot from three independent experiments is shown. The result of densitometric analysis is expressed as the ratio of phosphorylated protein amount to whole protein amount or of nuclear protein amount to cytosolic protein amount. Data are represented as mean ± SEM of three independent experiments. * *p* < 0.05, ** *p* < 0.01, or *** *p* < 0.001 against control (LPS positive/CLE negative) by Dunnett’s test.

We next conducted immunoblot analysis using RAW 264.7 cells to investigate the mechanism underlying the anti-inflammatory activity of CLE. LPS activates macrophages to upregulate mitogen-activated protein kinase (MAPK) and NF-κB pathways, leading to gene expression of various proinflammatory proteins [13,14]. CLE-treated RAW 264.7 cells were stimulated with LPS for 15 min, and the cell lysate was subjected to immunoblot analysis. As shown in Figure 6, phosphorylation of c-Jun N-terminal kinase (JNK), p38 MAPK, and extracellular signal-regulated protein kinases 1/2 (ERK1/2) was significantly enhanced by LPS stimulation. In addition, translocation of NF-κB to the nucleus was also induced following LPS stimulation. Conversely, phosphorylation of JNK and p38 MAPK in CLE-treated cells was significantly downregulated compared to non-CLE-treated cells, while ERK phosphorylation was not affected by CLE treatment. CLE treatment also significantly inhibited the nuclear translocation of NF-κB. These data indicated that gene expression of proinflammatory proteins induced by LPS is inhibited by CLE through downregulation of JNK, p38 MAPK, and NF-κB pathways.

### 2.7. Effect of CLE on Cytokine Levels in a Mouse Model of LPS-Induced Systemic Inflammation

The anti-inflammatory effect of CLE was further investigated in vivo using a mouse model of LPS-induced systemic inflammation. Mice were randomly assigned to three groups: control, LPS, and LPS + CLE. LPS + CLE group was orally administered CLE, while control and LPS groups received water as a vehicle for 7 consecutive days, and animals were weighed daily. There was no significant difference in body weight among all groups (Figure S1), suggesting that CLE has no apparent toxicity to mice. On day 7, mice were intraperitoneally injected with LPS to induce systemic inflammation. Two hours later, the blood, liver, and spleen were collected after euthanasia. There was no significant difference in the liver and spleen indices among all groups (Figure S1), suggesting that CLE was not toxic to either organ.

**Figure 7:**
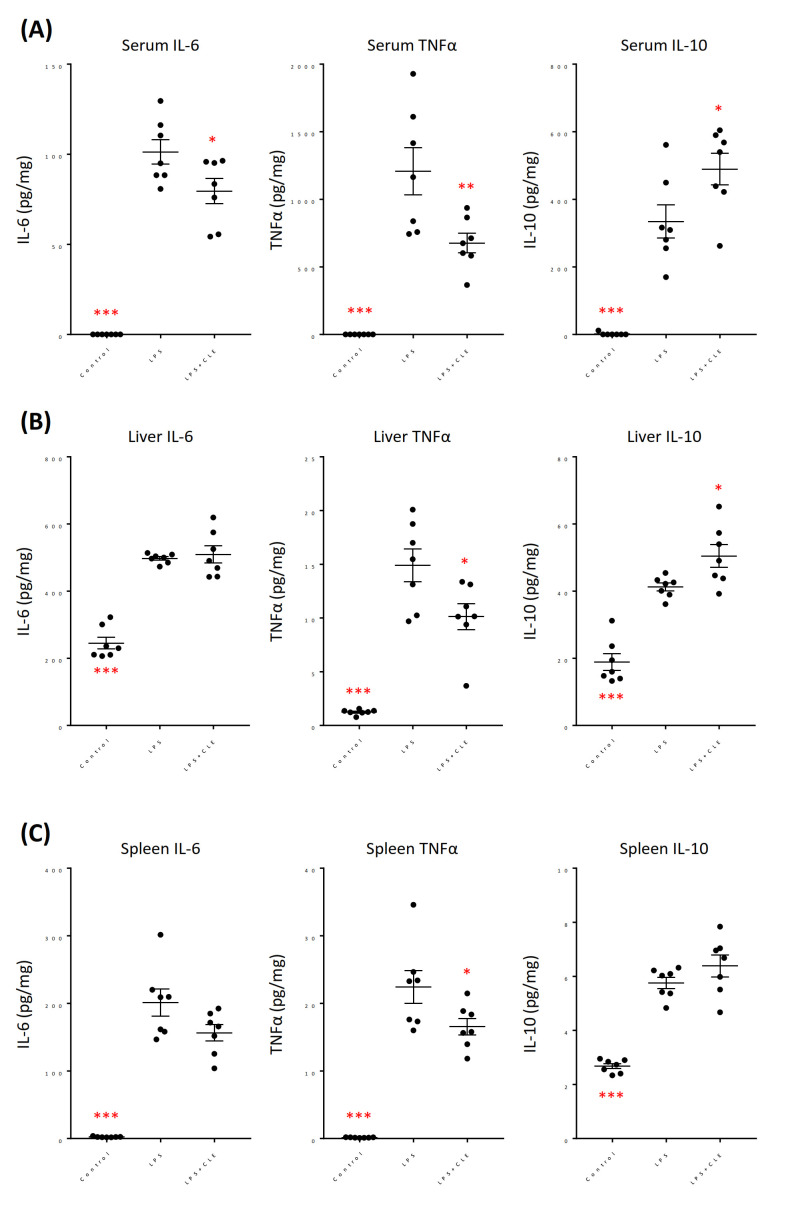
Effect of an aqueous extract from leaves of *Citrus unshiu* (CLE) on the cytokine levels in LPS-induced systemic inflammation mice. Control and LPS groups received water for 7 consecutive days, while LPS + CLE group received CLE (300 mg/kg). LPS and LPS + CLE groups were intraperitoneally injected with LPS (5 mg/kg) 2 h after the last oral administration on day 7, whereas control group was injected with 200 µL of PBS. Two hours later, all mice were anesthetized, and the cytokine levels in the blood, liver, and spleen were measured by ELISA. Data are represented as mean ± SEM (*n* = 7). * *p* < 0.05, ** *p* < 0.01, or *** *p* < 0.001 against LPS by Dunnett’s test. Serum (**A**), liver (**B**), spleen (**C**).

Serum cytokine levels of LPS-induced systemic inflammation model mice were measured. The data revealed that serum levels of IL-6, TNFα, and IL-10 significantly in-creased following LPS injection (Figure 7A). Conversely, oral administration of CLE significantly reduced the serum level of proinflammatory cytokines IL-6 and TNFα and increased that of an anti-inflammatory cytokine IL-10 compared with those of the LPS group. In the liver, all cytokines tested were significantly increased by LPS-induced systemic inflammation (Figure 7B). The TNFα level of LPS + CLE group significantly decreased in the liver compared with that of LPS group, whereas the IL-6 level of LPS + CLE group was similar to that of LPS group. As seen in the blood, CLE administration increased the IL-10 level in the liver. In the spleen, LPS injection induced a significant increase in all cytokine levels examined in this study (Figure 7C). The spleen IL-6 level of LPS + CLE group tended to decline compared with that of LPS group (*p* = 0.0586). The spleen TNFα level was significantly lower in LPS + CLE group than in LPS group, as observed in the serum and liver. Different from the data obtained from the blood and liver, there was no significant difference in the spleen IL-10 level between LPS and LPS + CLE groups.

### 2.8. Effect of CLE on Gene Expression of Inflammation-Related Proteins in LPS-Induced Systemic Inflammation Model Mice

**Figure 8:**
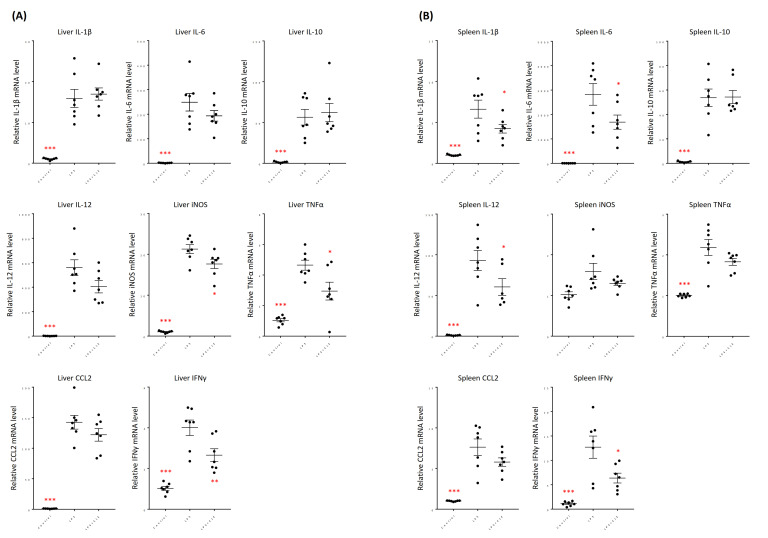
Effect of an aqueous extract from leaves of *Citrus unshiu* (CLE) on gene expression of inflammation-associated proteins in LPS-induced systemic inflammation mice. Control and LPS groups received water for 7 consecutive days, while LPS + CLE group received CLE (300 mg/kg). LPS and LPS + CLE groups were intraperitoneally injected with LPS (5 mg/kg) 2 h after the last oral administration on day 7, whereas control group was injected with 200 µL of PBS. Two hours later, all mice were anesthetized, and the liver and spleen were collected for real-time RT-PCR. Data are represented as mean ± SEM (*n* = 7). * *p* < 0.05, ** *p* < 0.01, or *** *p* < 0.001 against control by Dunnett’s test. Liver (**A**), spleen (**B**).

The expression levels of inflammation-associated genes in the liver and spleen of LPS-induced systemic inflammation model mice were examined. Almost all genes tested significantly increased in the liver and spleen after induction of systemic inflammation (Figure 8). In the liver, the expression levels of iNOS, TNFα, and interferon γ (IFNγ) genes were significantly lower in LPS + CLE group than in LPS group (Figure 8A). In addition, oral administration of CLE tended to reduce the IL-12 expression level (*p* = 0.0570) com-pared with that in the LPS group. Conversely, there was no significant difference in gene expression levels of IL-1β, IL-6, IL-10, and CCL2 between LPS and LPS + CLE groups.

In the spleen, oral administration of CLE significantly reduced gene expression levels of IL-1β, IL-6, IL-12, and IFNγ compared with those of LPS group (Figure 8B). Gene ex-pression levels of TNFα and CCL2 tended to decrease in LPS + CLE group compared with those of LPS group (*p* = 0.1082 and 0.1154, respectively). There was no significant difference in gene expression levels of IL-10 and iNOS between LPS and LPS + CLE groups.

### 2.9. Flavonoids in Samples

To characterize the flavonoid components in *C. unshiu* leaf used in this study, we quantified the main flavonoids by HPLC. As shown in Table 1, the quantitative analysis showed that the *C. unshiu* leaf contains polymethoxyflavones including nobiletin, tangeretin, 3,5,6,7,8,3′4′-heptamethoxyflavone, and sinensetin and flavanones including hesperidin and narirutin. Next, we analyzed the flavonoid contents in CLE. HPLC analysis of CLE revealed that only hesperidin was quantified among flavonoids tested. The concentration of hesperidin was 77 µM in 1.25 mg/mL of CLE. These data suggest that the glycosylated form of flavonoids can be extracted in water.

## 3. Discussion

In this study, we evaluated the anti-inflammatory effect of CLE, an aqueous extract from leaves of *C. unshiu*. The data shown in Figure 2A revealed the anti-inflammatory effect of CLE in which CLE significantly inhibited the production of two major proinflammatory cytokines IL-6 and TNFα and of a chemokine CCL2 by LPS-stimulated RAW 264.7 cells. The anti-inflammatory effect of *C. unshiu* peel has been demonstrated using LPS-stimulated RAW 264.7 cells thus far [9]; however, the anti-inflammatory effect of the aqueous extract from *C. unshiu* leaf has not been reported yet. This study is thus the first report demonstrating the anti-inflammatory effect of *C. unshiu* leaf.

CLE also significantly inhibited the production of IL-6 and tended to suppress the production of TNFα and CCL2 by primary peritoneal macrophages (Figure 2B). Differences in the degree of the inhibitory activity of CLE on the production of cytokines and chemokines between RAW 264.7 cells and mouse peritoneal macrophages may be at-tributed to different gene expression levels of the target molecule of CLE or of signaling molecules responsible for the production of inflammation-related cytokines and chemokines, such as suppressor of cytokine signaling-1 [17]. Decreased production of proinflammatory cytokines and CCL2 resulted from reduced expression of the genes as shown in Figure 4. Decreased production of nitric oxide (Figure 3) was also confirmed by reduced gene expression of iNOS (Figure 4).

Gene expression of proinflammatory cytokines in macrophages is regulated through NF-κB and MAPK pathways [18]. Reduced gene expression of IL-1β, IL-6, TNFα, CCL2, and iNOS, shown in Figure 4, seemed to be caused by downregulated activation of NF-κB by CLE treatment (Figure 6) because NF-κB is involved in gene expression of those inflammation-related proteins in macrophages [19,20]. In addition, decreased phosphorylation of p38 MAPK and JNK, shown in Figure 6, seemed to result in downregulated gene expression of IL-1β, IL-6, and TNFα (Figure 4) because these two kinases are related to gene expression of proinflammatory cytokines [21,22,23,24]. Overall results indicate that the bioactive substance in CLE may interact with the target molecule locating upstream of NF-κB and MAPK pathways to downregulate these intracellular signaling, leading to suppressed gene expression of inflammation-related proteins and thereby to decreased production of these gene products.

The anti-inflammatory effect of CLE was also evaluated in vivo using a mouse model of LPS-induced systemic inflammation. TNFα level in the serum, liver, and spleen was significantly decreased by CLE (Figure 7). Serum IL-6 level was also significantly reduced by CLE. These results are consistent with the in vitro data, suggesting that the bioactive substance in CLE can be absorbed into the mouse body to exert the anti-inflammatory activity. CLE significantly increased the level of IL-10, an anti-inflammatory cytokine, in the serum and liver. A ginsenoside Rg6, which is known to have a strong anti-inflammatory activity, has also been reported to enhance the IL-10 secretion in LPS-induced sepsis model mice [25]. As is the case with Rg6, the enhanced production of IL-10 will be responsible for the anti-inflammatory effect of CLE.

Gene expression profile also revealed that CLE inhibits the expression of inflammation-related cytokines such as IL-1β, IL-6, and TNFα in vivo (Figure 8). In addition, CLE also inhibited gene expression of other proinflammatory cytokines, IL-12 and IFNγ. IL-12 is known to regulate IFNγ production in cooperation with TNFα and to be required for lethality in a mouse model of endotoxic shock [26]. CLE may interact with dendritic cells to suppress IL-12 secretion from the cells to attenuate LPS-induced systemic inflammation responses [27].

To clarify the chemical property of CLE, component analysis was conducted using *C. unshiu* leaf. As shown in Table 1, the contents of flavanones in *C. unshiu* leaf used in this study were lower than those in *C. unshiu* fruits reported by Sun et al. [15]. Conversely, the contents of polymethoxyflavones in *C. unshiu* leaf were similar to those in *C. unshiu* fruits reported by Ortuño et al. [16]. We also measured the contents of flavanones and polymethoxyflavones in CLE. As a result, we quantified 47.3 µg/mL (77 µM) of hesperidin in 1.25 mg/mL of CLE and were not able to quantify other polymethoxyflavones or flavanones. Administration of hesperidin has been reported to attenuate LPS-induced endotoxicity in mice [28] and rats [29] and LPS-induced acute lung injury in mice [30,31]. In addition, hesperidin has been reported to ameliorate LPS-induced hepatic dysfunction and oxidative stress in rats [32]. Thus, the anti-inflammatory in vivo effect of CLE can be partially attributed to hesperidin as an anti-inflammatory bioactive molecule, because our results shown in this study are consistent with the data reported in these papers.

By contrast, the anti-inflammatory in vitro effect of CLE will not be attributed to hesperidin, because we did not observe the suppressive activity on proinflammatory cytokine production by LPS-stimulated RAW 264.7 cells in 77 µM hesperidin, which is the hesperidin concentration contained in 1.25 mg/mL of CLE (data not shown). However, hesperetin, an aglycon form of hesperidin, is a well-known anti-inflammatory agent [33] and has been reported to inhibit secretion of TNFα, IL-6, and IL-1β to reduce gene ex-pression of iNOS and to downregulate the NF-κB signaling pathway in RAW 264.7 cells [34]. Because hesperidin is cleaved by gut microorganisms to form hesperetin, and then hesperetin is absorbed into the body [35], the anti-inflammatory in vivo effect of CLE may be attributed to hesperetin converted from hesperidin. Hesperetin has been reported to be released into the bloodstream in form of glucuronide and sulphate conjugates after absorption [36]. Thus, the bioactive form of hesperidin in vivo may be hesperetin or its conjugates.

Other than flavanones and polymethoxyflavones, another possible bioactive sub-stance in CLE is a water-soluble peptide. Noh et al. reported an anti-inflammatory cyclic peptide isolated from the fruits of *C. unshiu* [37]. A low-molecular-weight polysaccharide is also a possible bioactive substance in CLE. Shin et al. reported an immunomodulatory polysaccharide isolated from the peels of *C. unshiu* [38]. At this moment, we have not identified the anti-inflammatory substance in CLE; further study is required to identify the water-soluble anti-inflammatory substance in *C. unshiu* leaf.

## 4. Materials and Methods

### 4.1. Reagents

Dulbecco’s modified Eagle’s medium (DMEM), RPMI 1640 medium, penicillin, streptomycin, fetal bovine serum (FBS), a protease inhibitor cocktail, and LPS from *Escherichia coli* 026/B6 were purchased from Sigma-Aldrich (St. Louis, MO, USA). Horseradish peroxidase-labeled anti-rabbit IgG antibody and rabbit monoclonal antibodies against ERK1/2, phosphorylated ERK1/2 (p-ERK1/2), JNK, phosphorylated JNK (p-JNK), p38 MAPK, phosphorylated p38 MAPK (p-p38 MAPK), NF-κB p65, lamin B1, and glyceraldehyde-3-phosphate dehydrogenase (GAPDH) were purchased from Cell Signaling Technology (Danvers, MA, USA). All other chemicals were purchased from Fujifilm Wako Pure Chemical (Osaka, Japan) or Nacalai Tesque (Kyoto, Japan) unless otherwise noted.

### 4.2. Preparation of C. unshiu Leaf Extract

Dried fine powder of *C. unshiu* leaf was provided by Ikata Service Inc. (Ikata, Ehime, Japan). The powder was soaked in water at 0.1 g/mL, stirred for 24 h at 12 °C, and centrifuged at 12,000× *g* for 20 min at 4 °C. The supernatant was then ultrafiltrated using a membrane with molecular weight cut-off of 1000. The filtrate was lyophilized and reconstituted with water. After adjusting pH to 7.4 with 1 M NaOH, the concentration was adjusted to 20 mg/mL. The extract was then filtered using a 0.22 μm membrane for sterilization and used for subsequent experiments as an aqueous extract from *C. unshiu* leaf (CLE).

### 4.3. Cells

Mouse macrophage-like cell line RAW 264.7 cells were obtained from European Collection of Authenticated Cell Cultures (ECACC, London, UK) and cultured in DMEM supplemented with 100 U/mL of penicillin, 100 μg/mL of streptomycin, and 10% FBS at 37 °C under humidified 5% CO_2_ in air. Mouse peritoneal macrophages were prepared as previously described [39] with modifications. In brief, 8- to 10-week-old female BALB/c mice (Clea Japan, Tokyo, Japan) were peritoneally injected with 3% thioglycollate medium (3 mL per mouse). Four days after injection, mice were sacrificed and injected with 3 mL of phosphate-buffered saline (PBS, pH 7.4) into the peritoneum to harvest thioglycollate-elicited peritoneal macrophages. After centrifugation at 200× *g* for 5 min at 4 °C, the collected cells were suspended in 1 mL of hemolysis buffer (155 mM NH_4_Cl, 15 mM NaHCO_3_, 1 mM ethylenediaminetetraacetic acid, pH 7.3). After adding 9 mL of PBS, the cell suspension was centrifuged at 200× *g* for 5 min at 4 °C. The cell pellet was then resuspended in RPMI 1640 medium supplemented with 100 U/mL of penicillin, 100 µg/mL of streptomycin, and 10% FBS and cultured in 60 mm culture dishes. After incubation at 37 °C under humidified 5% CO_2_ in air for 2 h, unattached cells were removed by washing with PBS twice. The attached cells were then scraped and used as peritoneal macrophages.

### 4.4. Cell Viability

Cytotoxicity of CLE to RAW 264.7 cells and mouse peritoneal macrophages was examined using a Cell Count Reagent (Nacalai Tesque) based on WST-8 according to the manufacturer’s instructions. Peritoneal macrophages and RAW 264.7 cells were seeded into a 96-well culture plate at 5.0 × 10^3^ cells/well and precultured at 37 °C for 2 and 16 h, respectively, under humidified 5% CO_2_. After removing the medium, peritoneal macro-phages and RAW 264.7 cells were cultured in the medium containing various concentrations of CLE for 24 h. After removing the medium, the cells were stimulated with 1.0 µg/mL of LPS in the medium for 6 h at 37 °C. After washing with PBS, fresh medium containing 10% Cell Count Reagent was added to each well, and the absorbance was measured at 450 nm using an iMark microplate reader (Bio-Rad Laboratories, Hercules, CA, USA).

### 4.5. Cytokine Immunoassay

The concentration of IL-6 was measured by an ELISA kit obtained from BioLegend (San Diego, CA, USA) according to the manufacturer’s instructions. The concentrations of TNFα, IL-10, and CCL2 were measured by ELISA kits obtained from eBioscience (San Diego, CA, USA) according to the manufacturer’s instructions. Mouse peritoneal macrophages and RAW 264.7 cells were seeded at 6.0 × 10^4^ cells/well and at 3.0 × 10^4^ cells/well, respectively, into 96-well culture plates. After preculturing of peritoneal macrophages and RAW 264.7 cells for 2 and 16 h, respectively, the cells were cultured in the medium containing various concentrations of CLE at 37 °C for 24 h. After removing the medium, the cells were stimulated with 1.0 µg/mL of LPS in the medium for 6 h at 37 °C. The culture medium was subsequently used for measuring cytokine concentrations. As for mouse tissues, 10 mg of the spleen and liver were homogenized in 0.5 mL of PBS containing a protease inhibitor cocktail and used for measuring cytokine concentrations.

### 4.6. Griess Assay

Griess assay was performed using a Griess Reagent System kit (Promega, Madison, WI, USA) according to the manufacturer’s instructions. RAW 264.7 cells were seeded into a 24-well culture plate at 2.0 × 10^4^ cells/well and precultured at 37 °C for 16 h. After re-moving the medium, the cells were cultured in the medium containing various concentrations of CLE at 37 °C for 24 h. After removing the medium, the cells were stimulated with 1.0 µg/mL of LPS in the medium for 24 h at 37 °C. The culture medium was subsequently used for measuring nitric oxide concentration.

### 4.7. Real-Time RT-PCR

RAW 264.7 cells were seeded into a 24-well culture plate at 1.0 × 10^5^ cells/well and precultured at 37 °C for 16 h. After removing the medium, the cells were cultured in the medium containing various concentrations of CLE at 37 °C for 24 h. After removing the medium, the cells were stimulated with 1.0 µg/mL of LPS in the medium for 24 h at 37 °C. The cells were then used for isolating total RNA using Sepasol-RNA I Super G (Nacalai Tesque) according to the manufacturer’s instructions. cDNA was synthesized with MMLV-reverse transcriptase (Promega) and an oligo-(dT)_11_ primer (Toyobo, Osaka, Ja-pan). Real-time PCR was performed using Thunderbird SYBR qPCR Mix (Toyobo), 5.0 pmol of a forward primer, 5.0 pmol of a reverse primer, and 1.0 μg of cDNA as previously described [40] with modifications. Thermal cycling conditions were 20 s at 95 °C, and 40 cycles of 3 s at 95 °C and 30 s at 60 °C. PCR products were measured on a StepOnePlus Real-time PCR System (Applied Biosystems, Foster City, CA, USA), and relative gene expression was calculated based on the comparative CT method using StepOne Software v2.1 (Applied Biosystems). β-Actin gene expression was used as an endogenous control. Specific oligonucleotide sequences for each gene are shown in Table 2.

### 4.8. Phagocytotic Activity

Phagocytotic activity was determined as previously described [39] with modifications. RAW 264.7 cells were seeded into a 24-well culture plate at 1.5 × 10^5^ cells/well and precultured at 37 °C for 16 h. After removing the medium, the cells were cultured in the medium containing 1.25 mg/mL of CLE at 37 °C for 24 h. After removing the medium, the cells were stimulated with 1.0 µg/mL of LPS in the medium for 1 h at 37 °C. After washing with PBS, the cells were cultured in the medium containing 100 μg/mL of Texas Red-conjugated Zymosan A (*Saccharomyces cerevisiae*) BioParticles (Molecular Probes, Eugene, OR, USA) at 37 °C for 24 h in darkness. After washing with PBS twice, the cells were scraped. After centrifugation at 200× *g* for 5 min at 4 °C, the cells were suspended in PBS containing 2% FBS and subjected to flow cytometric analysis using a FACSVerse flow cytometer (Becton Dickinson, Franklin Lakes, NJ, USA).

### 4.9. Immunoblot Analysis

RAW 264.7 cells were seeded into a 6-well culture plate at 1.0 × 10^6^ cells/well and precultured at 37 °C for 16 h. After removing the medium, the cells were cultured in the medium containing 1.25 mg/mL of CLE at 37 °C for 24 h. After removing the medium, the cells were stimulated with 1.0 µg/mL of LPS in the medium for 15 min at 37 °C. Cytosolic and nuclear proteins were then prepared using a CelLytic NuCLEAR Extraction Kit (Sigma-Aldrich) according to the manufacturer’s instructions. Immunoblot analysis was conducted as previously described [41]. Bands were visualized using a ChemiDoc XRS Plus apparatus (Bio-Rad Laboratories). The chemiluminescent intensity was quantified using the Quantity One software (Bio-Rad Laboratories).

### 4.10. A Mouse Model of LPS-Induced Systemic Inflammation

BALB/c mice were obtained from Clea Japan and housed in an animal room under a 12 h light/dark cycle at 24 ± 1 °C. Animals received a pelleted basal diet and water ad libitum. Systemic inflammation was induced according to our previous study [42] with modifications. After acclimatization to their housing environment for 1 week, 8-week-old female BALB/c mice were randomly assigned to 3 groups (7 mice per group): control and LPS groups received water by gavage (7.5 mL/kg) for 7 consecutive days (day 1 to day 7); LPS + CLE group received CLE by gavage (300 mg/kg) for 7 consecutive days. Animals were weighed daily. LPS and LPS + CLE groups were intraperitoneally injected with LPS (5 mg/kg) 2 h after the last oral administration on day 7, whereas the control group was injected with 200 µL of PBS. Two hours later, all mice were anesthetized, and the blood was collected by cardiac puncture to characterize inflammatory cytokine profiles. The liver and spleen were quickly removed, flash-frozen in liquid nitrogen, and kept at −80 °C until analysis. The study was conducted according to the guidelines of Animal Experiments of Ehime University and approved by the Animal Experiment Committee of Ehime University.

### 4.11. HPLC Analysis

For the analysis of flavanones and polymethoxyflavones, 500 mg of fine powder of *C. unshiu* leaf was soaked in 5 mL of dimethyl sulfoxide (DMSO)/methanol (1:1) in an ultrasonic bath for 10 min. After leaving for 1 h, the suspension was centrifuged at 10,000× *g* for 10 min and 9 volumes of water was added to the supernatant. A Bond Elut C18 cartridge (500 mg, Agilent Technologies, Santa Clara, CA, USA) was preconditioned with 3 mL of methanol and 6 mL of 10% aqueous methanol. The extract was then loaded into the cartridge, and the cartridge was washed with 15 mL of 10% aqueous methanol. Flavanones and polymethoxyflavones were eluted with 5 mL of DMSO/methanol (1:1) and filtered with a 0.22 μm PTFE membrane filter. HPLC analysis was performed at 40 °C on a BEH C18 column (2.1 × 100 mm, 1.7 μm, Waters, Milford, MA, USA). The gradient of mobile phase was acetonitrile in 10 mM ammonium formate aqueous solution as follows: remained at 30% for 4 min, 30–40% in 1 min, remained at 40% for 1 min, 40–55% in 1 min, remained at 55% for 3 min, at a flow rate of 0.25 mL/min. UV detection was performed at 230 nm.

For the analysis of flavanones and polymethoxyflavones in CLE, CLE was filtered with a 0.22 μm nylon membrane filter, and HPLC analysis was performed at 40 °C on a BEH C18 column (2.1 × 100 mm, 1.7 μm, Waters). The gradient of mobile phase was acetonitrile in 0.1% formic acid aqueous solution as follows: remained at 5% for 1 min, 5–95% in 10 min, remained at 95% for 1 min, at a flow rate of 0.4 mL/min. UV detection was performed at 230 nm.

### 4.12. Statistical Analysis

Data obtained were expressed as mean ± SEM. Statistical analysis was performed using GraphPad Prism version 7.05 (GraphPad Software, La Jolla, CA, USA). Statistical significance was determined via one-way ANOVA followed by Dunnett’s multiple comparison test as indicated. Values with *p* < 0.05 were considered statistically significant.

## 5. Conclusions

The anti-inflammatory effect of an aqueous extract from *C. unshiu* leaf was assessed in vitro and in vivo in this study. We found that CLE inhibits secretion of proinflammatory cytokines and a chemokine from RAW 264.7 cells and mouse peritoneal macrophages and of nitric oxide from RAW 264.7 cells. The inhibitory activity of CLE seemed to be at-tributed to downregulated JNK, p38 MAPK, and NF-κB signaling pathways, leading to suppressed gene expression of inflammation-associated proteins. Conversely, CLE did not affect the phagocytotic activity of LPS-induced RAW 264.7 cells. Oral administration of CLE significantly decreased the production of proinflammatory cytokines and increased that of an anti-inflammatory cytokine IL-10 in LPS-induced systemic inflammation mice. Thus, *C. unshiu* leaf is effective in attenuating inflammatory responses in vivo.

## Figures and Tables

**Table 1:** The contents of flavanones and polymethoxyflavones in *Citrus unshiu* leaf and fruit.

Compound		Contents in Leaf (mg/g (Dry Weight))	Contents in Fruit (mg/g (Dry Weight))
Flavanone	Narirutin	0.127 ± 0.006	43.90 ^1^
	Naringin	N.D.	N.D. ^1^
	Hesperidin	10.8 ± 0.0	261.63 ^1^
Polymethoxyflavone	Sinensetin	0.0225 ± 0.0003	0.017 ^2^
	Nobiletin	0.223 ± 0.007	0.182 ^2^
	Heptamethoxyflavone	0.0396 ± 0.0011	0.088 ^2^
	Tangeretin	0.0706 ± 0.0028 ^1^	0.054 ^2^

N.D. indicates not detectable. ^1^ Data are from Sun et al. [15]. ^2^ Data are from Ortuño et al. [16].

**Table 2:** Sequences of primers for real-time RT-PCR.

Target	Forward (5′-3′)	Reverse (5′-3′)
β-Actin	CATCCGTAAAGACCTCTATGCCAAC	ATGGAGCCACCGATCCACA
CCL2	CCACTCACCTGCTGCTACTCAT	TGGTGATCTTGTAGCTCTCC
IL-1β	AAGCCAGAGTCCAGAGAGAT	TTGGATGGTCTTGGTCCTTAGC
IL-6	AAGCCAGAGTCCTTCAGAGAGAT	TTGGATGGTCTTGGTCCTTAGC
iNOS	CCAAGGCCTCACCTACTTCC	CTCTGAGGGCTGACACAAGG
TNFα	CTACTCCCAGGTTCTCTTCAA	GCAGAGAGGAGGTTGACTTTC

## Data Availability

The data that support the findings in this study are available from the corresponding author upon reasonable request.

## Supplementary Materials

The following are available online at https://www.mdpi.com/article/10.3390/plants10081708/s1, Figure S1: Body weight, liver index, and spleen index of LPS-induced systemic inflammation mice.
