# Supplementary material for: Aqueous Extract from Leaves of Citrus unshiu Attenuates Lipopolysaccharide-Induced Inflammatory Responses in a Mouse Model of Systemic Inflammation
Source: Plants (Basel). 2021 Aug 19;10(8):1708. doi: 10.3390/plants10081708 (PMC8399385; doi:10.3390/plants10081708)
Supplement: Supplementary file 1 [file plants-10-01708-s001.zip › plants-1337646-SI.pdf]

Supplementary Figure

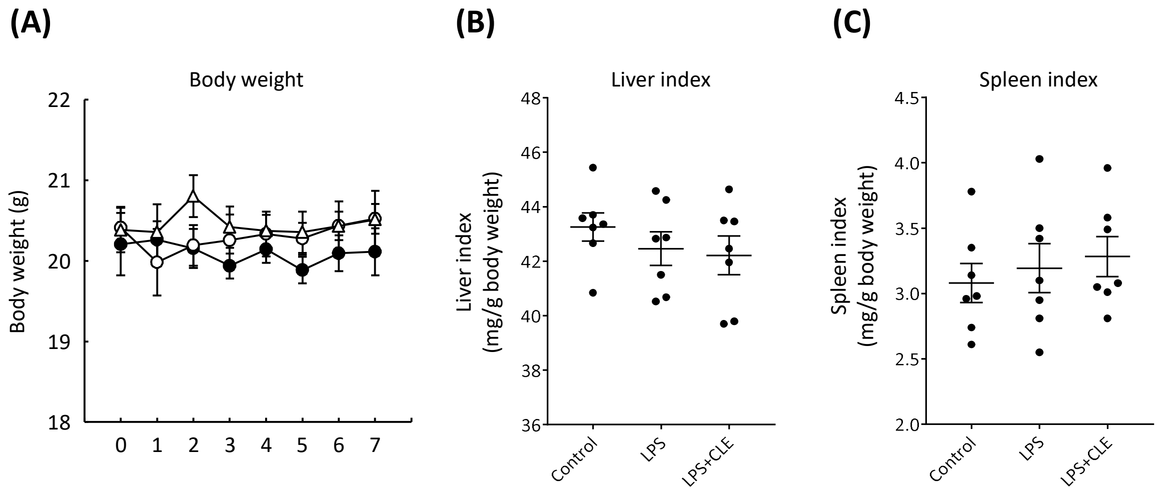

Figure S1. Body weight, liver index, and spleen index of LPS-induced systemic inflammation mice. Control and LPS groups received water for 7 consecutive days, while LPS+CLE group received CLE (300 mg kg^{-1}). LPS and LPS+CLE groups were intraperitoneally injected with LPS (5 mg kg^{-1}) 2 h after the last oral administration on day 7, whereas control group was injected with 200 μL of PBS. Two hours later, all mice were anesthetized, and the liver and spleen were weighed. Data are represented as mean ± SEM (n = 7). (A) Body weight was measured daily during oral administration of CLE. (B) The liver weight was measured after euthanasia. (C) The spleen weight was measured after euthanasia. There is no statistically significant difference against LPS group by Dunnett's test.
